# Current Situation and Perspectives of Clinical Study in Integrative Medicine in China

**DOI:** 10.1155/2012/268542

**Published:** 2012-02-21

**Authors:** Jie Wang, Xingjiang Xiong

**Affiliations:** Department of Cardiolorgy, Guang′anmen Hospital, China Academy of Chinese Medical Sciences, 5 Bei Xian Ge Street, Xi Cheng District, Beijing 100053, China

## Abstract

Integrative medicine is not only an innovative China model in clinical practice, but also the bridge for TCM toward the world. In the past thirty years, great achievements have been made in integrative medicine researches, especially in clinical practice. The clinical achievements mainly include the following three: innovating methodology of disease-syndrome combination, excavating the classical theory in traditional Chinese medicine (TCM), preventing and curing refractory diseases. The development ideas and strategies of integrative medicine for future mainly include (a) standing on frontier field of international medicine and improving the capability of preventing and curing refractory diseases; (b) moving prevention and control strategy forward and improving the curative effect of common and frequent disease; (c) excavating the classical theory of TCM and broadening the treatment system of modern medicine; (d) improving the innovation level of new high effective drugs on the basis of classical prescriptions and herbs in TCM; (e) rerecognizing the theory of formula corresponding to syndrome in TCM and enhancing the level of clinical research evidence based on evidence-based medicine. Integrative medicine will do obtain greater achievements in creating new medicine and pharmacology and make more tremendous contributions for the great rejuvenation of the Chinese nation and human health care.

## 1. Introduction

The coexistence of western medicine (WM) and traditional Chinese medicine (TCM) began to appear when WM was introduced to China from the middle of 16th century. The tendency of “confluence of Chinese and western medicine” appeared as the two medical systems contacting and influencing with each other since then. With the development of modern medical technology, intercourse, and cooperation between TCM and WM, integrative medicine was established in the 1980s. Under the guidance of “system learning, comprehensively mastering, sorting, and improving,” predecessors of integrative medicine have been exploiting the complementary advantages of macro and micro, global and local, structure and function, traditional and modern, disease differentiation, and syndrome differentiation in WM and TCM, in order to create new medicine and pharmacology theory. Through unremitting efforts of integrative medicine staffs at home and abroad, remarkable achievements have been made in health care, teaching, researching, academic development, discipline construction, talent training, and so forth. So we can say that integrative medicine is not only an innovative China model in clinical practice, but also the bridge for TCM toward the world [1–3]. Now the clinical achievements of the past 30 years and developing strategies of integrative medicine are described as follows.

## 2. Clinical Achievements

### 2.1. Innovating Methodology of Disease-Syndrome Combination: A New Mode for Syndrome Research

The relationship between disease and syndrome is thought to be one of the most significant problems in TCM clinical and basic practice. As a new mode for syndrome research, disease-syndrome combination mainly refers to absorbing the idea and theory of disease differentiation in western medicine (WM) as well as syndrome differentiation in TCM. The mode, which originates from the medical practice since more than half a century ago, has realized mutual compensation of advantages of TCM and WM [1, 2]. Combining whole thinking, imagery thinking, and dialectical thinking in TCM with materialism of modern medical sciences, the mode can be regarded as a good cut-in point and successful control pattern for integrative medicine [3]. It has complementary advantages of WM and TCM and marks a new era created by integrative medicine in clinical researches. Seminar on the academician Chen Keji's academic thinking about “the new mode of disease-syndrome combination and its application in clinical practice” was held in Beijing on May 23, 2011. Professor Chen and his students discussed the scientific connotation of this new mode and its application in diagnosis, treatment, and scientific research together. China news of traditional Chinese medicine, a famous domestic media, made follow-up report on the symposium, which had evoked large repercussions.

Academician Chen Keji pointed out that modern view of disease-syndrome combination includes six aspects: (a) mode of disease differentiation by WM combined with syndrome differentiation by TCM; (b) mode of syndrome differentiation and treatment combined with specific prescription for certain illness; (c) mode of treating according to disease staging; (d) mode of differentiation of the basic pathogenesis combined with syndrome differentiation and treatment in TCM; (e) mode of treating according to syndrome differentiation rather than disease differentiation when there's no disease can be diagnosed in WM; (f) mode of treating according to disease differentiation rather than syndrome differentiation when there's no syndrome can be diagnosed in TCM [4]. The emphasis of the mode could be played on either syndrome or disease. As the connotation of syndrome in TCM is significantly different from disease in WM, laying special emphasis on syndrome means that syndrome is just the basis of therapeutic scheme. On the contrary, effective therapeutic plans should be formulated according to disease differentiation when special emphasis is laid on disease. This new mode is beneficial to the original innovation in diagnosis and treatment. The advantages of the mode include four aspects as follows. (a) *Definitely diagnosing*. As the disease diagnosis in TCM is vague and extensive, it is entirely necessary and possible to absorb some relevant achievements of disease diagnosis in WM for definitely diagnosing. (b) *Targeted treating*. As the new mode pays more attention to the therapeutic evaluation of disease, it could achieve more definite therapeutic targets and stable curative effect compared with syndrome differentiation mode alone. (c) *Accurately prognosing*. Summarization of clinical phenomena is the principal judgment basis for prognosis in TCM, therefore, the prognosis judgment is always not very accurate. However, the new mode has vital guidance value for treatment and prognosis judgment. (d) *Deepening classics*. Due to the succinctness and conciseness of TCM classics, the essential features of the disease and syndrome could be rerecognized and deepened through combining with the modern cognition of pathology, diagnostics and pharmacology study in WM.

Syndrome is not only the core of TCM basic theory and syndrome differentiation, but also the bridge to associate disease and formula. Different from diagnosis based on pathological mechanism, syndrome is a classification according to subjective symptom and objective sign collected by physical examination [5–7]. Premise studies on disease-syndrome combination lies in syndrome diagnostic criteria and therapeutic evaluation system. Researches on syndrome diagnostic criteria aim at establishing the scientific and normative diagnosis system, while researches on therapeutic evaluation aim at constructing an objective evaluation system. Under the leadership of academician Chen Keji, we are the first to study and report on blood stasis syndrome in coronary heart disease based on the new mode. Contributed to the diagnosis of blood stasis syndrome in coronary heart disease, 19 items such as precordial pain, dark purple tongue color, and erythrocyte deformability were selected based on the calculation analysis of 48 kinds of examination items in 92 cases patients with coronary heart disease. And the clinical diagnosis accordance rate was 89%. 6 items giving the greatest contribution to diagnosis such as blood viscosity and total cholesterol (TC) were confirmed by a stepwise regression analysis for 21 items such as hemorheology and blood lipid [8]. Correlation analysis of blood stasis syndrome and pathological changes shown in coronary angiography with coronary heart disease showed that the blood stasis syndrome score was significantly correlated to the maximal stenosis degree and coronary lesion score demonstrated by coronary angiography before percutaneous coronary intervention (PCI), and the correlation was increased along with the increasing of the patients' age and the course of the disease. Conclusions were also verified in our related researches [9–11].

Through mathematical statistics method and computational intelligence approach, it was found out that the major syndrome factors of coronary heart disease are blood stasis, qi deficiency, turbid phlegm, qi stagnation, heat deposition, yang deficiency, yin deficiency, and cold coagulation based on calculation analysis of 5099 cases patients reported on literatures and 1069 cases patients with coronary heart disease validated by coronary angiographic. We also constructed the diagnosis scales of blood stasis syndrome and its accompanied syndromes in coronary heart disease, such as qi deficiency and blood stasis syndrome and qi stagnation and blood stasis syndrome [12–14]. In the study of therapeutic evaluation system, taking coronary heart disease as example, important indexes such as syndrome evaluation scale, clinical critical events, and quality of life were selected on the basis of completely evaluating the present indexes through application of clinical epidemiology-/evidence-based medicine method. Meanwhile, high validity and reliability of therapeutic evaluation system of coronary heart disease was constructed through comprehensive analysis of various index by the hall for workshop of metasynthetic engineering. Clinical efficacy scale of TCM syndrome and the primitive entry pool of scale for patient-reported outcomes of coronary heart disease were established by our team [15].

### 2.2. Excavating the Classical Theory in TCM

It is meaningful to promote the original innovation in integrative medicine researches through further understanding the connotation of syndrome diagnosis, therapeutic principle, and classical prescription by modern science and technology. Among these studies, three researches below are honored.

The first one is blood stasis syndrome theory and the clinical application of the method of promoting blood circulation and removing blood stasis. Blood stasis syndrome theory is first recorded in *The Songs of Chu*, a classical literature written in ancient China. *Shuo Wen Jie Zi *(Text Notes and Word Explanations) written by Xu Shen in the Eastern Han Dynasty explained that blood stasis is hematocele. And it was frequently mentioned in TCM classics such as *the Canon of Internal Medicine*, *Treatise on Febrile Diseases*, and *Synopsis of Golden Chamber*. Inspired by the prominent TCM doctor Guo Shikui's experience in treating angina pectoris by Decoction for Removing Blood Stasis, a classic formula developed by Wang Qingren in Qing Dynasty, academician Chen Keji advocated treating coronary heart disease by activating blood circulation to dissipate blood stasis principally. His team was the first to study the diagnostic criteria of blood stasis syndrome and report the quantitative scoring method, which had been extensively used in domestic and was the first study in using objective quantitative method in TCM syndrome study. Objectified study of abdomen diagnosis on blood stasis syndrome was also superior to the research methods of Japan in the same period. Standards of syndrome differentiation and therapeutic evaluation of coronary heart disease were formulated according to above-mentioned researches, which had already become national standards. The essence of blood stasis syndrome and mechanism of treating coronary heart disease by activating blood circulation to dissipate blood stasis had been elucidated at various levels of intact animal, tissue, cell, molecule, and gene protein expression. As coronary restenosis after coronary artery balloon injury and stent placement have been considered an international difficult problem, academician Chen firstly treated it by decoction for removing blood stasis and optimized the prescription to a more simplified and effective recipe, *Xiong Shao *Capsule (XSC). A randomized controlled trial (RCT) about XSC showed that the restenosis rate in XSC group treated by XSC on the basis of routine western therapy was decreased by 45% compared with routine western therapy group, and the experimental studies showed that XSC could suppress the gene expression of proliferation of vascular smooth muscle cells [16–19]. With important academic value and clinical significance, the study has promoted the academic development of TCM greatly, which had been awarded the first award of national science and technology progress in 2003.

The second study is the theory of dispelling interior pathogenic factors and purgation and its application in the treatment of acute abdomen. According to the theoretical basis of “the six fu-viscera function well when unobstructed,” academician Wu Xianzhong began to explore integrative medicine therapy on acute abdomen in early years. Through unifying standard for syndrome differentiation and defining operative indication, his team accumulated a large number of valid acute abdomen cases with the therapeutic method of expelling pathogens by purgation. Under the leadership of Professor Wu, the multidisciplinary and prospective researches included the effects of dispelling interior pathogenic factors and purgation on the splanchnic blood flow and caecal single smooth muscle cell, the clinical characteristics of multiple organ dysfunction syndrome (MODS) caused by several different kinds of elements, the changes of nerve-endocrine-immunological network in MODS and the effects of purgative herbs on information transmission mechanism of immune cells through four times of different scale joint research in the seventh to tenth five-year plan period. After years of efforts, significant progress had been made and the operation rate had been reduced in the treatment of acute abdomen, such as severe acute cholangitis, acute severe pancreatitis, and complicated biliary stones, which was awarded the second award of national science and technology progress in 2003 [20–22].

The third one is the theory of “treating the toxifying disease with poisonous agents” and researches for arsenic trioxide (AS_2_O_3_) treating acute promyelocytic leukemia (APL). The theory is a traditional simple understanding of hypertoxic drug treating difficult and complicated diseases. It was recorded in *Compendium of Materia Medica* that the herbal nature of arsenolite is very hot and poisonous, while white arsenic sublimed from arsenolite is more poisonous. White arsenic is a traditional external drug for removing the necrotic tissue and promoting granulation plaster, the effective component of which is AS_2_O_3_. The research was enlightened by the prominent TCM doctor's experienced external prescription for treating skin cancer. On the basis of verification of curative effects and optimizing prescription, researchers developed the arsenous acid injection from the experienced external prescription, which had definitive curative effect for the patients with APL and reached the top level in the world [23–26]. The mechanism of arsenous acid treating APL was illustrated from the perspective of molecular oncology, including degradation of PML/RARa fusion proteins, downregulating gene expression of Bcl2 and inducing apoptosis in leukemia cells. Arsenous acid became the first antileukemia drug of inducing apoptosis in the world arousing the medical research fever of arsenic trioxide [27–29]. It was honored as “ancient remedy performs new tricks” in 1996 by Science [30]. Sloan-Kettering and his coworkers reported that 12 patients with recurrence of APL after conventional chemotherapies were treated with AS_2_O_3_, and 11 cases of them relieved completely in 1996. This paper, published in New England Journal of Medicine, directly led to the widely acceptance of AS_2_O_3_ in the treatment of APL in the international medical field [31].

### 2.3. Preventing and Curing Refractory Diseases

As the frontier field and hot issue of cardiovascular diseases, restenosis after percutaneous coronary intervention and myocardial ischemia reperfusion injury (MIRI) during open heart surgery of cardiopulmonary bypass has become the best innovative points of clinical studies in integrative medicine. Researches showed that restenosis after percutaneous coronary intervention was closely related to blood stasis syndrome. Predominantly evaluated by restenosis (RS) rate estimated by coronary angiography (CAG), a prospective randomized controlled study was carried out on RS after PCI to observe the intervention effect of *Xiong Shao* Capsule (XSC). Compared with the control group, the incidence of RS rate in the XSC group was significantly lower (24.1% versus 48.5%, *P* < 0.05) and the extent of angiostenosis and diameter of the culprit arteries, determined by CAG, also significantly reduced after patients had been treated for 6 months with [(2.21 ± 0.85) mm versus (1.72 ± 0.99) mm, *P* < 0.05], and [(26.58 ± 20.72) % versus (41.19 ± 30.92) %, *P* < 0.05], respectively. The incidence of clinical end-point event was significantly lower in the XSC group than that in the control group (11.7% versus 27.6%) and the *P* value was close to statistical significance (*P* = 0.051). Comparing with the control group, the blood-stasis syndrome score in the XSC group was also significantly lower (*P* < 0.01). The results showed that XSC had a wide range of therapeutic effects including effectively preventing RS after PCI in combination with conventional western medical treatment, decreasing the attack of angina pectoris and improving the blood stasis syndrome. Experimental researches on blood activating herbs showed that it can significantly inhibit pathological vascular remodeling after balloon injury, thus reduce late lumen loss and prevent restenosis [32–36].

As the establishing the cardiopulmonary bypass of open heart surgery is key point of successful operation, myocardial ischemia reperfusion injury (MIRI), which is very obvious during the recovery of circulation, has become the hot issue needed to be resolved. Some scholars found that the pathogenesis of MIRI during open heart surgery of cardiopulmonary bypass is deficiency of heart qi in the origin and excess of heart blood stasis and internal turbid toxin in the superficiality and the therapeutic principles are boosting qi and nourishing heart, activating blood circulation and resolving toxin simultaneously. It was proposed that of astragalus injection and tetramethylpyrazine injection for boosting qi and activating blood circulation should be given by vein injection during operation and *Hu Xin Bao* (compatibility of extracts of ginseng and panax notoginseng with taurine) for boosting qi, activating blood circulation, and resolving toxin should be given by oral administration before operation. The research showed that astragalus injection combined with tetramethylpyrazine injection could reduce the content of MDA and myocardial enzymes' release and improve the activity of SOD, NO, and NOS. Serial studies demonstrated that boosting qi combined with activating blood circulation have significantly synergetic effects, and boosting qi, activating blood circulation, combined with resolving toxin were superior to those simple boosting qi, activating blood circulation, resolving toxin, and boosting qi combined with activating blood circulation [37, 38].

Multiple organ dysfunction syndrome (MODS) is one of the difficult problems in the field of the critical care medicine, which is characterized by acute onset, rapid progress, and extremely high mortality. Since the 1970s of 20th century, some scholars began to take vigorous action to explore a new way of preventing and treating MODS by integrative medicine and a new theory of “bacteria and bacterial toxin treated simultaneously” was presented ultimately. They also perfected schemes for the diagnosis procedure and treatment standard of MODS by both TCM and integrative medicine. And four therapeutic principles for the main types of syndromes were put forward, such as activating blood circulation to dissipate blood stasis therapy on blood stasis syndrome, clearing heat and toxin therapy on heat toxin syndrome, reinforcing the vital energy and consolidating the constitution therapy on acute deficient syndrome, and dispelling interior pathogenic factors and purgation therapy on Yangming fu-organ syndrome. Integrative medicine therapy can effectively improve the clinical efficacy and shorten the course of the disease thus reducing mortality. A famous injection of Chinese medicine, “*Shen Nong* 33,” with the effect of activating blood circulation to dissipate blood stasis and antiendotoxin, was developed, which has reduced the mortality of international recognized infectious four or more organs failure from 100% to 50% and reached the international advanced level. Furthermore, a new strategy of “bacteria, bacterial toxin, and inflammatory mediator treated simultaneously” was put forward on the basis of the theory of “bacteria and bacterial toxin treated simultaneously.” *Xue Bi Jing *injection, the first Chinese medicine preparation in emergency medicine, was developed, which have made great contributions to the advancement of critical care medicine [39–43].

Chronic hepatitis B is the common disease in China, as well as in the world, causing great affliction to patients. It has become the major issue in the treatment of chronic liver disease. The progression of chronic hepatitis B may lead to liver cirrhosis and hepatocellular carcinoma. Hepatic fibrosis is the common pathological end stage of various chronic liver diseases regardless of the etiology, and blocking the occurrence and development of fibrosis of liver is very important in chronic hepatic diseases' treatment and prognosis. TCM has become the important therapy in treating chronic hepatitis, liver fibrosis, and liver cirrhosis. Some scholars put forward the hypothesis that liver fibrosis and early liver cirrhosis can be reversed. They found out that the basic pathogenesis of liver fibrosis is weakened body resistance and blood stasis, so therapeutic method of strengthening body resistance and dispelling stasis was established, and “*Fu Zheng Hua Yu *Capsules,” a new drug for treating liver fibrosis, was developed. Predominantly evaluated by liver tissue fibrosis, clinical researches were carried out to observe the curative effect of the therapeutic method of strengthening body resistance and dispelling stasis. The total inversion rate of liver tissue fibrosis was 52% to 58.3% compared before and after treatment, which also confirmed that liver fibrosis can be reversed and treated. The mechanism includes significantly inhibiting lipid peroxidation, the proliferation of hepatic stellate cell and activation of collagen expression, reducing inflammation of hepatocytic injury model, increasing the activity of matrix metalloproteinases, promoting the degradation of pathological liver collagen, and so on [44–46].

Combining the macroscopic view with microscopic view, syndrome differentiation with disease differentiation, regional with global, taking stopgap measures with taking radical measures, supporting healthy aspects with eliminating pathogens, tumor treatment model by integrative medicine emphasizes contriving individual treatment plan and evaluation standard on the basis of biological characteristics and the course of disease. Malignant tumors could be treated by TCM therapies such as reinforcing the vital energy and consolidating the constitution, supplementing qi and nourishing yin, and clearing away heat and toxic materials, combined with conventional therapies such as radiotherapy, chemotherapy, and surgery. TCM treatment has significances in decreasing toxicity and increasing efficacy on radiotherapy and chemotherapy. Integrative medicine theory has a remarkable effect in alleviating symptoms such as dry mouth in hyperpyrexic consumption of yin syndrome and deficiency of both qi and yin syndrome caused by head and neck cancer after radiotherapy, relieving symptoms such as cough caused by acute radiation pneumonitis, improving immune function, and survival quality of postoperative patients, preventing the tumor from recurrence or metastasis and prolonging survival time. The new model of combining TCM and modern cancer treatment has attracted widespread attention in the world, which is known as “China Model for Cancer Treatment” [47]. In addition, screening of tumor inhibition from more than 3,000 species of Chinese herbs and nearly 300 Chinese herbal compound, effective components having directly killing effect on cancer cell such as indirubin, camptothecin, vinblastine, matrine, and aclitaxel were extracted. Some Chinese herbs, having the effect of immunological enhancement and biological response modifier-like action such as polyporus, poria cocos, and mushroom, were also found out.

APL is a special type of acute leukemic (AL). TCM suggests that the pathogenesis of APL is weakened body resistance and excessiveness of pathogen, so therapeutic method of eliminating pathogenic factors and strengthening body resistance was established. Some scholars developed the Compound Realgar Natural Indigo Tablets (Realgar, Indigo Naturals, Salvia and Radix pseudostell) on the basis of clearing away heat and toxic materials and supplementing qi with activating blood circulation and promoting hemogenesis method. 155 cases of APL patients were treated by the Compound Realgar Natural Indigo Tablets and the remission rate was 97.42% after treating for 6 months. No side effect, serious infection, bleeding, and DIC were found during the treatment course. It was also characterized by higher negative conversion rate of PML—RAR*α* fusion gene and simple application. The results demonstrated that the complete remission rate of treatment of the Compound Realgar Natural Indigo Tablets were 10–15% higher than that of all-trans retinoic acid (ATRA). On this basis, the effect of post-remission therapy mainly with Compound Realgar Natural Indigo Tablets on long-term survival of 74 cases patients with APL showed that the median remission time was 48 months with recurrence rate only 14.86% and 10-year survival probability was 75.38% [48–50].

Since the 1970s of 20th century, the basic syndrome of type 2 diabetes included yin deficiency with internal excessive heat, deficiency of both qi and yin, and deficiency of both yin and yang, therefore, III-type differentiation of type 2 diabetes was established and developed. It had already been adopted by national guidelines for new drug in the late 1980s. As deficiency of both qi and yin was the important basic syndrome of the disease, “*Jiang Tang Jia *tablets,” a new Chinese herb of supplementing qi and nourishing yin, could improve insulin resistance, islet **β**-cell function, and the level of glucose and lipid metabolism, the total effective rate of which was 76.54%. In addition, researches of *Tang Wei Kang* capsule treating early diabetic nephropathy and *Tang Xin Ping* treating diabetic cardiopathy have gotten progress [51, 52]. Some scholars also found out that blood stasis was another significant pathogenesis of type 2 diabetes due to the changes of hemorheology with different degree were found. So they advocated treating the disease by promoting blood circulation and removing blood stasis principally. Based on this idea, promoting blood circulation by removing blood stasis recipes, such as nourishing yin and activating blood recipes and *Xian Zhen* tablet of reinforcing kidney and activating blood, were developed. Those recipes have multilevel and multitarget effects, including improving symptoms, reducing blood glucose, improving blood rheology and blood flow, lowering triglycerides (TGs), and malondialdehyde (MDA), enhancing activity of erythrocyte SOD, Na^+^-K^+^-ATP enzyme and Ca^2+^-Mg^2+^-ATP enzyme, and so forth. The experimental studies showed that the effect of *Xian Zhen* tablet includes lowering blood glucose and glycosylated hemoglobin, decreasing urine protein excretion, improving renal function, reducing the pathological changes of glomerular mesangial expansion and basement membrane thickening, decreasing AGEs amounts of renal cortex, and downregulating RAGE-mRNA expression in renal cortex and endothelia of heart vessel. It provided a new idea for preventing and treating diabetic and chronic vascular complications [53, 54].

Severe pancreatitis, namely, acute hemorrhagic necrotizing pancreatitis, is characterized by acute onset, rapid progress, high mortality, and poor prognosis. 65% of the death cases are due to complicating with acute respiratory distress syndrome (ARDS). According to the theoretical basis that “the six fu-viscera function well when unobstructed” and “the lung and the large intestine are interior-exteriorly related,” acute pancreatitis is treated by expelling pathogens by purgation, and the average cure rate reached to 97%, while the average cure rate of severe pancreatitis was 80%. Compared with our country and abroad, the mortality has reached the lowest level. *Qing Yi *decoction, a famous antipyretic and purgative prescription, protected the lung from injury in many aspects, by preserving the damage of gut barrier function, reducing or eliminating endotoxemia derived from the gut, inhibiting the production, and release of TNF, IL-6, and the translocation of bacteria. The results may fully show the superiority of integrative medicine in treating serious diseases [55, 56].

A certain progress was also made on dermatosis and burn medicine by integrative medicine therapy. Vitiligo was effectly treated by taking modified *Tao Hong Si Wu *decoction, external application of compound tar traditional Chinese rubbing-drugs and melagenine extracted from placenta. 243 patients with vitiligo were treated by modified *Tao Hong Si Wu* decoction and the total effective rate was 68.2%, the mechanism of which was related to upregulation of tyrosinase activity, increasing the melagenine content, and promoting melanocyte proliferation [57]. Moist exposed burn therapy (MEBT), a new therapeutic system of burn medicine in integrative medicine, has become the leading enabler throughout the world. It is found out that the burn wound should be kept in a moist but not macerated environment in order to promote in nature recovery and generation of the skin rather than in traditional dry environment. And the exact curative effect was obtained by MEBT and moist exposed burn ointment (MEBO) [58].

Severe acute respiratory syndrome (SARS) has aroused international attention for strong infectiousness, rapid progression, poor prognosis, and high mortality, which has no special effective therapy yet. 524 patients of SARS in China were divided into integrative medicine treatment group (*n* = 318) and western medicine treatment group (*n* = 206). The existence rates for the symptoms of weakness, short breath, dyspnea in the first group were significantly lower than that in the second group after treatment. The duration of weakness was averagely shortened by 1.5 days in the first group. And short breath, dyspnea, and muscle aching pain were averagely shortened by 2 days, 1 day, and 2 days, respectively. Researches showed that the effect of integrated therapy of TCM and WM for treating SARS was superior to WM treatment alone, and the integrative medicine could improve clinical symptoms such as weakness, short breath, and dyspnea [59–61]. The exact clinical curative effect was also recognized by World Health Organization (WHO).

## 3. Developing Strategies

### 3.1. Standing on Frontier Field of International Medicine and Improving the Capability of Preventing and Curing Refractory Diseases

Previous achievements in clinical researches of integrative medicine showed that it is absolutely necessary to keep a foothold at frontier field of international medicine and life science and derive the wisdom and new theories from these subjects in order to find the innovation and breakthrough from subject cross and osmosis. Aiming at the hot issues and knotty problems confronted in clinical medicine, we could put forward scientific hypotheses in exploring the etiology and pathogenesis of the disease and seek for the effective therapeutic principles and classical prescriptions. Basing on the research mentioned above, the clinical efficacy should be objectively evaluated by randomized controlled trials (RCT), and the potential mechanism should be illustrated ultimately. By summarizing the clinical regularity in time, it will contribute to the innovation of the medical theory and guide clinical practice.

Taking coronary heart disease as example, despite great advancements in the fields of basic and clinical researches made by modern medicine, there are still some issues to be resolved, such as acute coronary syndrome complicated by microvascular thrombosis, myocardial ischemia-reperfusion injury, no-reflow phenomenon, stent thrombosis, obvious subjective symptoms such as hypodynamia and shortness of breath remained after percutaneous coronary intervention (PCI), and ventricular remodeling following myocardial infarction [62–64]. Previous study showed that the prospects of integrative medicine is brightening in treatment for coronary stent thrombosis and protecting the myocardial ischemia-reperfusion injury.

In addition, as viral infectious disease belonged to the category of epidemic febrile disease in TCM thousands years ago, Chinese ancients had accumulated rich experience and formed a systematic and complete theory in treatment. Currently, better therapeutic efficacy of viral infectious diseases could be achieved by combining two medical systems, especially in SARS, N1H1, and bird flu. Also, more similar breakthrough points of integrative medicine can be found, for instance, improving the low success rate of assisted reproductive technology (ART) by combining ART with TCM therapeutic method of reinforcing kidney and activating blood, and so forth.

### 3.2. Moving Prevention and Control Strategy Forward and Improving the Curative Effect of Common and Frequent Disease

“Moving prevention and control strategy forward” is a national macrohealth policy, which well adapted to the new medical model, “physiological-psychological-social-environmental” model. It means that the focal point of medicine will be transferred from treating disease to health care, and disease prevention will be paid more attention to. Therefore, the policy of “prevention first” will be carried out instead of traditional ideological concept “treatment is more major than prevention.” It is similar to the TCM theory of “preventive treatment of disease,” including principles of “preventing measure taken before the occurrence of disease” and “preventing measure taken after the occurrence of disease” in *Canon of Internal Medicine*. Concrete measures of “moving prevention and control strategy forward” include concept forward, funding forward, emphasis of the researches forward, and measures to be carried out forward. It could reduce the incidence of the major diseases from the origin and effectively control the medical expense and save resources in medicine and health. Integrative medicine researches should also observe the principles above and pay more and more attention to improve the curative effect of common and frequent diseases.

Taking cardiovascular disease, for example, there are about 30% of the population in the world died from cardiovascular and cerebrovascular events, among which 62% of stroke and 49% of cardiovascular events were directly caused by hypertension [65]. According to the China cardiovascular reports (2008-2009), the occurrence and mortality of cardiovascular disease is still increasing in our country, and it is estimated that the number of patients with cardiovascular disease is at least 230 million. It also demonstrated that there were about 200 million hypertensive patients in China with more than 10 million patients increased annually. As the primary cardiovascular risk factor, the risk level of hypertension is equivalent to three other cardiovascular risk factors together. That is why more emphasis should be taken on prevention and intervention of earlier-stage hypertension in clinical researches of integrative medicine. Additionally, hyperlipidemia, hyperglycemia, obesity, and other risk factors also should be paid more attention to. It is reported by World Health Organization (WHO) that if risk factors were controlled as early as possible, 80% of the disease can be prevented effectively, such as coronary heart disease, stroke, and diabetes. Furthermore, paying 1* yuan* in prevention will save 7-8 *yuan *in treatment.

### 3.3. Excavating the Classical Theory of TCM and Broadening the Treatment System of Modern Medicine

JAMA, an international authoritative journal, have commented that traditional medicine should joint tracks with modern medicine. It suggested that not only should we inherit traditional academic thoughts but also keep an eye on modernization of TCM and study it in a scientific and systematic way [66]. The target is to fully digest the traditional Chinese medicine, and apply it to modern medical system. How to do it? The first is further understanding of the essence in TCM, while giving up the dross. The second is illustrating the mechanism of the traditional therapy by using advanced scientific technology in order to improve the safety of the treatment and alleviate the toxicity adverse effect.

Viscera, meridians, prescriptions, and syndromes, the precious wealth in TCM left by our ancients, are worth deeply researching into. However, as the theory is profound, classical, and concise, combining with clinical practice is the unique way to understand the connotation. For example, the lung and the large intestine are interior-exteriorly related, that is to say, the lung was associated with the large intestine by meridians. In the clinical practice, dysfunction of the large intestine conduction could cause no descending of the lung qi, conversely, no descending of the lung qi also can result in obstruction of fu-qi. It is reported that introducing fu-unblocking and purgation therapy into adults' acute pneumonia is a rapid and effective treatment. In the 70th of the last century, Professor Wang had applied *Liang Ge San* (Cool Diaphragm Powder) to treat SIRS and MODS, which mainly manifested as Yangming visceral substantive syndrome. The result demonstrated that the respiratory function of 80% patients with respiratory failure was rapidly improved, and the recovery was greatly promoted. Among the patients with acute pancreatitis accompanied with MODS, the therapy also received superior efficacy. All of these theories mentioned above, including blood stasis syndrome theory, theory of dispelling interior pathogenic factors and purgation, and theory of “treating the toxifying disease with poisonous agents,” were all worth deeply excavated, which have being greatly broadened the treatment system of modern medicine.

### 3.4. Improving the Innovation Level of New High Effective Drugs on the Basis of Classical Prescriptions and Herbs in TCM

Compared with TCM theory, Chinese herbs are much easier to be modernized and recognized. Therefore, it is of great significance in promoting modernization, industrialization, and industrialization of the Chinese herbs, by ways of combining modern technology with fully understanding of classical prescriptions and herbs.

China is a great power with rich herb resources. According to the records in Formula Dictionary of Traditional Chinese Medicine, there are approximately 100 thousand prescriptions, including special prescriptions and herbs for certain diseases. The classical prescriptions and herbs provided with definite clinical indications are of more meaning to be developed. That will provide an effective shortcut to improve the capability of developing new drugs, which is characterized by definite chemical structure, explicit action mechanism, obviously curative effect, advanced formulations, convenience for taking, and low price.

At present, the effective fractions and monomers extracted from Chinese herbs had obtained reliable clinical benefits. For instance, artemisinin extracted from Sweet Wormwood (*Artemisia annua L.*) have a definite effect in the treatment of falciparum malaria, which had been confirmed by multinational joint researches. The new therapy developed by Professor Tu Youyou has saved millions of lives across the globe, especially in the developing world, which had also been listed in the catalog of “essential medicines” by the World Health Organization (WHO) [67–71]. Therefore, Professor Tu was awarded the 2011 Lasker~DeBakey Clinical Medical Research Award for the discovering artemisinin and its utility for treating malaria. The research will raise a new global round of climaxes of modernization and internationalization of TCM.

In addition, indirubin extracted from indigo naturals in* Danggui Long Hui* Wan (Pill of Angelica sinensis, Gentian and Aloe) could treat leukemia. Diterpenonid versicolaction extracted from Tripterygium Wilfordii Hook can be used as immunosuppressive agents for treating rheumatoid arthritis (RA). Biphenyl dimethyl dicarboxylate (DDB) extracted from *Schisandra chinensis* could decrease the ALT and AST activity. Tetramethylpyrazine extracted from Ligusticum Chuanxiong Hort has showed a good effect for ischemic cerebrovascular disease. Cantharidin extracted from Mylabris could treat liver cancer. Moreover, other active components such as ginsenoside, total puerarin flavonoids, polyporus umbellate polysaccharides, ganoderma lucidum polysaccharide, anisodamine, tanshinone, trichosanthin, tetrahydropalmatine, tetrandrine, rubidate, ilexonin A, and ferulic acid sodium.

### 3.5. Rerecognizing the Theory of Formula Corresponding to Syndrome in TCM and Enhancing the Level of Clinical Research Evidence Based on Evidence-Based Medicine

Evidence-based medicine (EBM) is a new subject quickly developed in the clinical medicine field in the 1990s. The core thinking is to combine evidence, personal experiences, and patients' actual situation to formulate scientific measures for preventing diseases, promoting the recovery and improving life quality. Among them, clinical evidence originates mainly from randomized controlled trial (RCT), systematic review, and meta-analysis. With medical science transforming from traditional experience medicine into evidence-based medicine, fundamental changes have taken place in clinical medicine. Therefore, following the principle of respecting science and evidence, it is of the utmost importance to enhance the level of clinical research evidence in TCM and integrative medicine [72–74].

It is noteworthy that the treatment concept, formula corresponding to syndrome, lied in classical works of TCM, is similar to the ideas of EBM. However, it had been ignored for a long time. The theory of syndrome differentiation and formula corresponding to syndrome are two characteristic inheritance veins in TCM. Generally speaking, the former is always the mainstream ideology in TCM, while the latter has been paid little attention. Clinical medication based on pathogenesis is the core idea of syndrome differentiation, while clinical medication based on formula syndrome is not exactly the same as it. The most significant difference between them is whether giving attention to the objective evidences of formula utilization. The theory of formula corresponding to syndrome attaches great importance to the objective indications of herbs, which mainly comes from long-term, large-scale and repeated clinical trials by Chinese ancients.

As the indications of herbs are objective and concrete, clinical effect could be repeated at anytime, anywhere, and for anybody. So it is suggested that, facing with one patient, 10 TCM physicians may prescribe the identical prescription and get rapid treatment effect simultaneously according to the indications of formulae and herbs. This is just the reason why the significant curative effect can be by classical prescriptions get in treating severe and lingering illness. The extractive process of indications is similar to the evidence-based research, and indications of herbs have probably exceeded the category of expert experience in EBM. Therefore, carrying out clinical studies under guidance of EBM and formula corresponding to syndrome is helpful to summarize the indications of formulae and herbs, and enhance the level of clinical research evidence in TCM [75–79].

## 4. Summary

The history of man's science development showed that the crossing and blending of two kinds of knowledge systems will be able to set up a new knowledge system. Integrative medicine, an unprecedented task in present world, is a new pattern of medicine, which is formed by the integration of TCM and WM. The current situation of integrative medicine career was highly evaluated by academician Han Qide. He pointed out that integrative medicine is an inevitable choice for the development of Chinese medicine and the breakthrough point of development for modern medicine, which have unique advantages and will play an important role in China. With changing of the disease chart, increasing of metabolic disease, malignant tumor, iatrogenic disease and drug-induced disease, and the coming of senile society as well as the change of people's views on health and medical mode, both opportunities and challenges have been brought to the development of integrative medicine. Thus we believed that under the guidance of “pay equal attention to both WM and TCM” and “implementing the integrative medicine and developing TCM,” integrative medicine will obtain great achievements in creating new medicine and pharmacology, which builds on the combination of both WM and TCM, and make tremendous contributions for the great rejuvenation of the Chinese nation and human health care [80].
